# Parameters that affect outcome in surgical ICU patients

**DOI:** 10.1186/cc11012

**Published:** 2012-03-20

**Authors:** A Vakalos, M Petkopoulou, D Jannussis

**Affiliations:** 1 Xanthi General Hospital, Xanthi, Greece

## Introduction

Surgical ICU patients have a lower severity illness score on ICU admission day. The aim of our study was to compare the length of stay (LOS), ventilation days (VD) and parameters that affect the APACHE II-III scoring system between surgical patients who died in the ICU and surgical patients who survived and discharged from the ICU.

## Methods

During November 2005 and May 2011, 310 patients were admitted to our medical and surgical ICU. From these, 122 were surgical patients (39.35%) and were included retrospectively in our study. Mean age was 64 years, mean APACHE II score 14.5, actual mortality rate 12.29%. The patients were separated into two groups. Group A involved 107 surgical patients who survived the ICU and group B 15 surgical patients who died in the ICU. We looked for statistical significant difference (two-tailed *P *value) between the mean APACHE values at admission of group A and group B, using the unpaired Mann-Whitney test (nonparametric) or the unpaired *t *test Welch corrected (parametric), according to the normality test.

## Results

The mortality rate of surgical patients was 12.29%. We detected no statistical difference between the two groups according to age (*P *= 0.27), heart rate (*P *= 0.13), temperature (*P *= 0.57), Na (*P *= 0.44), K (*P *= 0.18), WBC (*P *= 0.56), Ht (*P *= 0.7), PaO_2 _(*P *= 0.28), PaCO_2 _(*P *= 0.7), albumin (*P *= 0.21), glucose (*P *= 0.68) and GCS (*P *= 0.26). We detected statistically significant higher group B values according to BUN (*P *= 0.015), creatinine (*P *= 0.005), bilirubin (*P *= 0.0032), APACHE II score (*P *= 0.0018), LOS (*P *< 0.0001) and VD (*P *< 0.0001). We detected statistically significant higher group A values according to mean arterial pressure (*P *= 0.0052) and PH (*P *= 0.0027).

## Conclusion

According to our data, surgical patients who died (group B) had higher severity score on admission. Nevertheless, the main difference between surgical patients who died and who survived the ICU was hemodynamic instability, which was severe enough to cause hypoperfusion, metabolic acidosis, early acute kidney injury and early multiple organ dysfunction. As a result, the length of stay and the ventilation days were higher in group B patients, assuming that early and effective surgical management is important in order to avoid early multiple organ dysfunction on ICU admission.

